# Curcumin as a Potent and Selective Inhibitor of 11β-Hydroxysteroid Dehydrogenase 1: Improving Lipid Profiles in High-Fat-Diet-Treated Rats

**DOI:** 10.1371/journal.pone.0049976

**Published:** 2013-03-22

**Authors:** Guo-Xin Hu, Han Lin, Qing-Quan Lian, Shu-Hua Zhou, Jingjing Guo, Hong-Yu Zhou, Yanhui Chu, Ren-Shan Ge

**Affiliations:** 1 School of Pharmacy, Wenzhou Medical College, Wenzhou, China; 2 The 2nd Affiliated Hospital, Wenzhou Medical College, Wenzhou, China; 3 Heilongjiang Key Laboratory of Anti-fibrosis Biotherapy, Mudanjiang Medical University, Mudanjiang, Heilongjiang, PR China; 4 Population Council, New York, New York, United States of America; Bioinformatics Institute, Singapore

## Abstract

**Background:**

11β-hydroxysteroid dehydrogenase 1 (11β-HSD1) activates glucocorticoid locally in liver and fat tissues to aggravate metabolic syndrome. 11β-HSD1 selective inhibitor can be used to treat metabolic syndrome. Curcumin and its derivatives as selective inhibitors of 11β-HSD1 have not been reported.

**Methodology:**

Curcumin and its 12 derivatives were tested for their potencies of inhibitory effects on human and rat 11β-HSD1 with selectivity against 11β-HSD2. 200 mg/kg curcumin was gavaged to adult male Sprague-Dawley rats with high-fat-diet-induced metabolic syndrome for 2 months.

**Results and Conclusions:**

Curcumin exhibited inhibitory potency against human and rat 11β-HSD1 in intact cells with IC_50_ values of 2.29 and 5.79 µM, respectively, with selectivity against 11β-HSD2 (IC_50_, 14.56 and 11.92 µM). Curcumin was a competitive inhibitor of human and rat 11β-HSD1. Curcumin reduced serum glucose, cholesterol, triglyceride, low density lipoprotein levels in high-fat-diet-induced obese rats. Four curcumin derivatives had much higher potencies for Inhibition of 11β-HSD1. One of them is (1E,4E)-1,5-bis(thiophen-2-yl) penta-1,4-dien-3-one (compound 6), which had IC_50_ values of 93 and 184 nM for human and rat 11β-HSD1, respectively. Compound 6 did not inhibit human and rat kidney 11β-HSD2 at 100 µM. In conclusion, curcumin is effective for the treatment of metabolic syndrome and four novel curcumin derivatives had high potencies for inhibition of human 11β-HSD1 with selectivity against 11β-HSD2.

## Introduction

**Figure 1:**
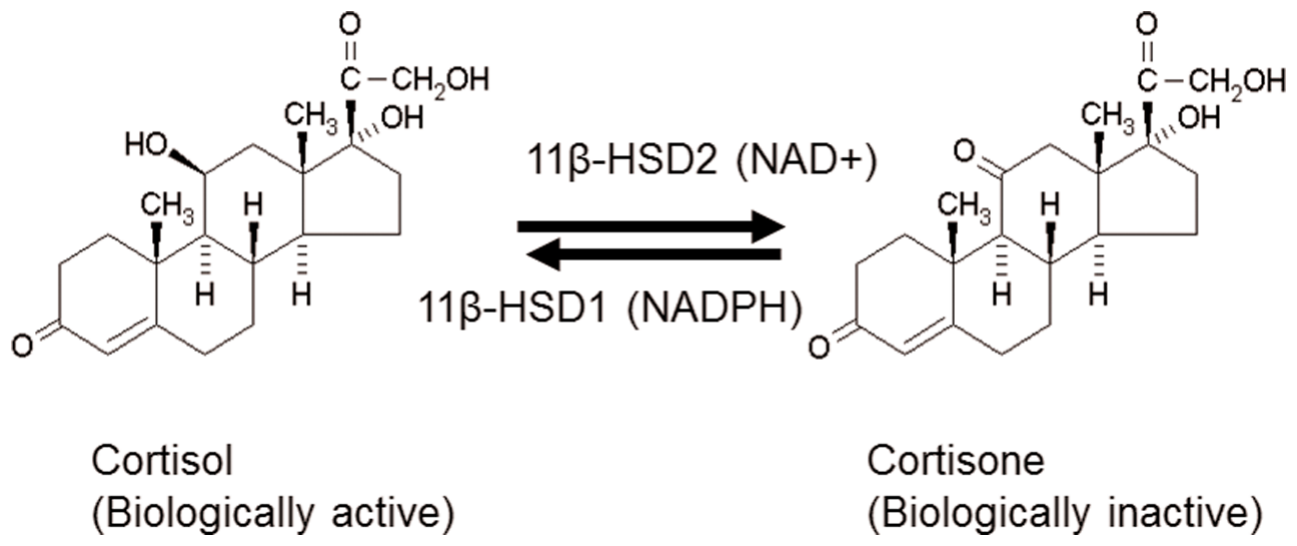
Interconversion of cortisol and cortisone by two 11β-hydroxysteroid dehydrogenase (11β-HSD) isoforms. 11β-HSD1 catalyzes the conversion of cortisone into cortisol in the liver or fat tissues, and 11β-HSD2 catalyzes the conversion of cortisol into cortisone in kidney or colon tissues.

Glucocorticoids (GCs) have a wide range of physiological and pharmacological roles in mammalian functions [1]. Excessive GCs under conditions such as stress and Cushing's syndrome cause a spectrum of clinical features, including metabolic syndrome [2]. GCs increase glucose output in the liver, induce fat accumulation, dampen glucose-dependent insulin sensitivity in the adipose tissue, thus increasing the risks of metabolic syndrome [3]. Intracellular levels of GCs (cortisol in the human or corticosterone, CORT, in the rat) are regulated by 11β-hydroxysteroid dehydrogenase (11β-HSD), which has two known isoforms: an NADP^+^/NADPH dependent 11β-HSD1 oxidoreductase that behaves a primary reductase in the liver and fat tissues (Fig. 1) and an NAD+ dependent 11β-HSD2 [4], [5]. 11β-HSD2 acts a unidirectional oxidase to prevent cortisol from stimulating the mineralocorticoid receptor in kidney and colon, and the mutation of human 11β-HSD2 gene (*HSD11B2*) causes severe hypertension and hypokalemia [5].

**Figure 2:**
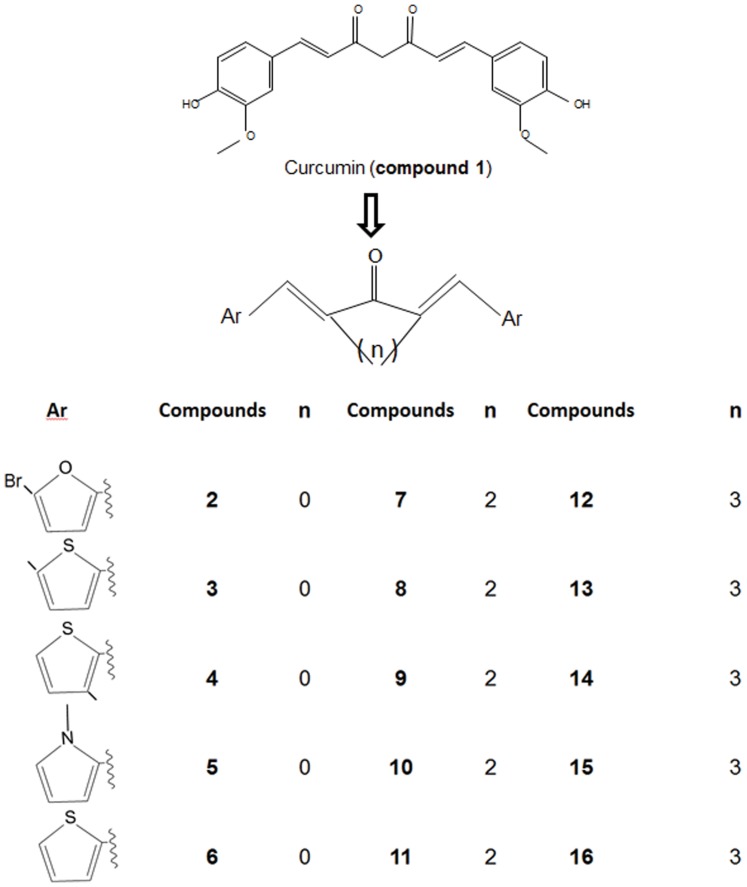
Structures of curcumin and its pentadienone analogues. Ar = aryl group; n = carbon numbers, where n = 0, designating open chain pentadienone compound, and n = 2, 3 designating cyclopentadienone compound.

Recently, 11β-HSD1 inhibition has gained attention as a potentially effective method for treating metabolic syndrome, including type 2 diabetes [6]. 11β-HSD1 knockout mice are resistant to diet-induced obesity and glucose intolerance, and 11β-HSD1 over-expression in the fat tissue causes metabolic syndrome [3], [7]. We screened many nutraceuticals to study whether they had inhibitory effects on 11β-HSD1 with selectivity against 11β-HSD2. One of chemicals is curcumin (Fig. 2, compound **1**). In this study, we investigated the therapeutic efficacy of curcumin for high-fat-diet (HFD)-induced metabolic syndrome in a rat model, and we also screened 12 curcumin analogues (Fig. 2) to test whether these compounds specifically inhibit 11β-HSD1 activity. It is important that 11β-HSD1 inhibitors should not significantly inhibit 11β-HSD2 in order to avoid undesirable sodium retention, hypokalemia, and hypertension.

## Materials and Methods

### Chemical and animals

[1,2,6,7-^3^H] Corticosterone (^3^H-CORT) and [1,2,6,7-^3^H] cortisol (^3^H-cortisol) were purchased from Dupont-New England Nuclear (Boston, MA). ^3^H-11Dehydrocorticosterone (^3^H-11DHC) and ^3^H-cortisone were prepared from labeled ^3^H-CORT or ^3^H-cortisol as described earlier [8]. Cold CORT, 11DHC, cortisol and cortisone were purchased from Steraloids (Newport, RI). Curcumin, icariin and berberine was purchased from Sigma-Aldrich Company (St Louis, MO, USA). The library of nutraceuticals was obtained from Wenzhou Medical College (Wenzhou, China). Human liver and kidney microsomes were purchased from Gentest (Woburn, MA). Male Sprague-Dawley rats (body weight 140–180 g) were purchased from Wenzhou Medical College Animal Center (Wenzhou, China) for HFD-induced metabolic syndrome treatment experiment. Male Sprague-Dawley rats (90 days old) were purchased from Charles River Laboratories (Wilmington, MA) for the isolation of Leydig cells for 11β-HSD1 enzyme inhibition assay. Both animal protocols were approved by the Institutional Animal Care and Use Committee of the Rockefeller University and Wenzhou Medical College.

### Chemistry

Curcumin analogues were synthesized by coupling the appropriate aldehyde with acetone, or cyclopentanone or cyclohexanone in an alkaline medium, respectively as described [9], [10]. The melting points and spectroscopic analysis such as NMR and mass spectroscopy were performed. The reaction was carried out with a rate 2∶1 of substituted aldehydes and ketones. For example, the reaction of 5-bromofuran-2-aldehyde with three respective ketones in alkaline medium was performed to generate these compounds as previously described [9], [10].

### Preparation of microsomes

Rats were euthanized by CO2, and livers, testes and kidneys were collected. Rat liver, testis and kidney microsomes were prepared as described previously [11]. In brief, rat liver and kidney were homogenized in 0.01 mM PBS buffer containing 0.25 M sucrose, and nuclei and large cell debris were removed by centrifugation at 1500× g for 10 min. The post-nuclear supernatants were centrifuged twice at 105,000×g, the resultant microsomal pellets were resuspended. Protein contents were measured by Bio-Rad Dye Reagent Concentrate (Cat.#500-0006). Microsomes were used for measurement of 11β-HSD1 and 11β-HSD2 activities.

### Rat Leydig cell isolation

Rat Leydig cells contain the highest 11β-HSD1 activity in all cell types of rat tissues [12], and were used for rat 11β-HSD1 enzyme sources. Purified rat Leydig cells were obtained from 90-day-old Sprague-Dawley rats by collagenase digestion of the testes followed by Percoll density centrifugation of the cell suspension, according to the previously described method [13]. Adult rat Leydig cells were harvested from the Percoll gradient at a band at 1.070 mg/ml. The purity of cell fractions was evaluated by histochemical staining for 3β-hydroxysteroid dehydrogenase activity with 0.4 mm etiocholanolone as the steroid substrate [14]. Enrichment of rat Leydig cells was typically more than 95%.

### Construction of expression human *HSD11B1* plasmid and transfection

An expression plasmid was constructed to express human 11β-HSD1 (*HSD11B1*) cDNA in pcDNA I expression vector from its original human *HSD11B1* vector (pBluescriptSK+).[15]. The *Escherichia coli* transformants carrying an insert were selected by colony hybridization, and a clone with the insert in the correct orientation relative to the vector T7 promoter was identified by restriction mapping. All transfections were carried out on 80% confluent cultures in 12-well plates. Aliquots of 1 µg *HSD11B1* pcDNA I were transfected into mammalian CHOP cells with the FuGENE Transfection Reagent (Roche) according to manufacturer's protocol. Cells were allowed to grow for 24 hours in media containing 10% fetal bovine serum. Then media were removed and cells were harvested for 11β-HSD1 activity assay.

### 11β-HSD1 assay in intact rat Leydig cells and CHOP cells transfected with *HSD11B1*

11β-HSD1 reductase activity was performed in intact cells using endogenous cofactor NADPH as described previously [16]. In brief, each assay tube contained 25 nM substrate 11DHC (for rat) or cortisone (for human), spiked with 30,000 cpm their respective ^3^H-11keto-steroid in the PBS buffer. 25×10^3^ cells were added to each tube to initiate the reaction and the reaction mixture was incubated for up to 2 hrs, during which the reaction is within the linear range. At the end of reaction, the reaction was stopped by adding 2 ml ice-cold ether. The steroids were extracted, and the organic layer was dried under nitrogen. The steroids were separated chromatographically on thin layer plates in chloroform and methanol (90∶10, v/v), and the radioactivity was measured using a scanning radiometer (System AR2000, Bioscan Inc., Washington, DC) as described previously [11]. The percentage conversion of 11DHC to CORT or cortisone to cortisol was calculated by dividing the radioactive counts identified as 11-OH-steroids by the total counts.

### 11β-HSD1 assay in rat and human microsomes

The rat testis and liver or human liver microsomes were used for 11β-HSD1 assay. 11β-HSD1 activity was performed in the microsome according to a previously described method [17]. In brief, the assay tubes contained 25 nM substrate 11DHC (for rat) or cortisone (for human), spiked with 30,000 cpm their respective ^3^H-11keto-steroid, 0.2 mM NADPH and 5 mM glucose-6-phosphaare in the PBS buffer. 2 µg microsomes were added to each tube to initiate the reaction and the reaction mixture was incubated for up to 2 hrs, during which the reaction is within the linear range. The rest procedure was similar to 11β-HSD1 assay in intact cells.

### 11β-HSD2 assay in rat and human kidney microsomes

The rat and human kidney microsomes were used for 11β-HSD2 sources. 11β-HSD2 activity was performed in the microsome according to a previously described method [17]. In brief, the assay tubes contained 25 nM substrate CORT (for rat) or cortisol (for human), spiked with 30,000 cpm their respective ^3^H-11β-hydroxyl steroid, 0.2 mM NAD+ and 0.1 mM DTT in the PBS buffer. 8 (rat) or 20 (human) µg kidney microsomes were added to each tube to initiate the reaction and the reaction mixture was incubated for up to 3 hrs, during which the reaction is within the linear range. The rest procedure was similar to 11β-HSD1 assay. The percentage conversion of CORT to 11DHC or cortisol to cortisone was calculated by dividing the radioactive counts identified as 11keto-steroids by the total counts.

### Determination of half maximum inhibitory concentrations (IC50) and inhibitory mode

The IC_50_ was determined by adding different concentrations of each compound in the 11β-HSD1 or 11β-HSD2 reaction as described [17]. The mode of inhibition was assayed by adding various concentrations of steroid substrates in the presence of an inhibitor as described [18].

### Animal treatment

Thirty male Sprague-Dawley rats (body weight 140–180 g) were randomly divided into three groups: vehicle control (normal diet), HFD and HFD plus 200 mg/kg curcumin, with 10 rats in each group. Rats were gavaged with vehicle (0.1% cellulose) in normal diet control and HFD groups, or with 200 mg/kg/day of curcumin (suspended in 0.1% cellulose) in the HFD plus curcumin group for two months. By the end of curcumin treatment, rats were euthanized. The body, liver, kidney and testis weights were recorded. Sera were collected after placing the bloods at room temperature for 25 min and centrifuged at 1500 g/min for 20 minutes. Sera were used for measurement of serum glucose, and lipid analysis.

### Serum glucose and lipid analysis

Serum glucose, total cholesterol, low density triglyceride (TG), lipoprotein (LDL), apolipoprotein A1 is (APOA1) and apolipoprotein B (APOB) were measured using a Hitachi 7600 biochemical analyzer (Hitachi, Japan) according to standard clinical protocol.

### Statistics

Enzyme data were subjected to nonlinear analysis by GraphPad (Version 5, GraphPad Software Inc., San Diego, CA) for IC_50_. Lineweaver-Burk plot was used for the mode of inhibition. Data were subjected to analysis by one-way ANOVA followed by Tukey's multiple comparisons testing to identify significant differences between groups when three were calculated. Differences were regarded as significant at P<0.05.

## Results

### Screening the selective inhibitors

**Figure 3:**
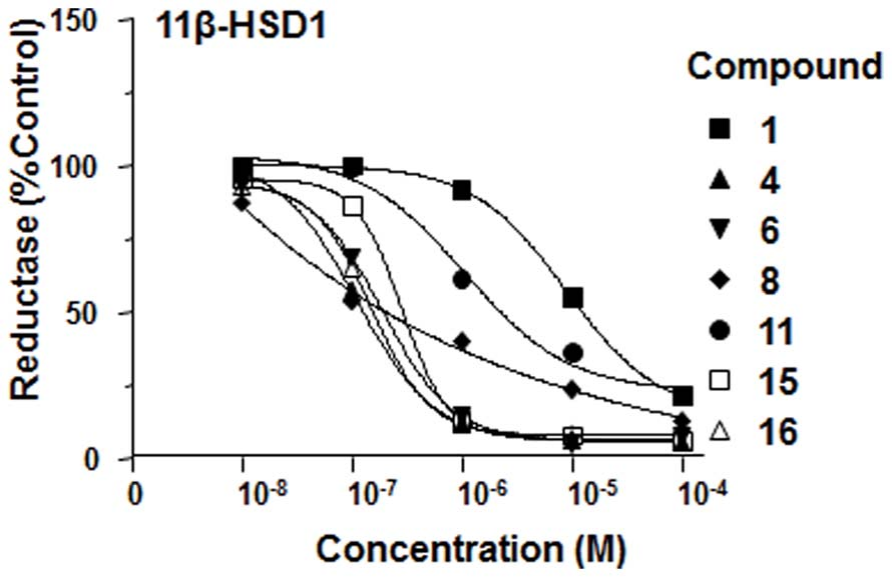
Dose-dependent inhibition on 11β-HSD1 in intact rat Leydig cells by curcumin (compound 1) and it derivatives.

Using microsomes from CHOP cells transfected with human *HSD11B1* and adult rat testis as 11β-HSD1 sources, we screened many nutraceuticals, including curcumin, icariin and berberine, and found that only curcumin (compound **1**) showed inhibitory effects against human and rat 11β-HSD1, with IC_50_ values of 10.62±7.17 µM and 4.18±0.24 µM, respectively. In intact CHOP cells transfected with human *HSD11B1* and adult rat Leydig cells, curcumin showed inhibitory effects against human and rat 11β-HSD1, with IC_50_ values of 5.78±2.22 µM and 2.29±0.69 µM, respectively, indicating that curcumin was slightly potent when the enzyme was assayed in intact cells. We further used intact cells to screen curcumin derivatives (Fig. 2). Thiophenyl 1,4-pentadiene-3-one compounds **4** and **6** were among the most potent inhibitors (Table 1 and Fig. 3). Compound **4** [(1E,4E)-1,5-bis(3-methylthiophen-2-yl) penta-1,4-dien-3-one] was 12.54 and 50.75 times more potent for the inhibition of human and rat 11β-HSD1 activity than curcumin, respectively (Table 1). Compound **6** [(1E,4E)-1,5-bis(thiophen-2-yl) penta-1,4-dien-3-one] was 24.68 (human) and 31.44 (rat) times more potent than curcumin, respectively (Table 1). There are clear structure-activity responses for these compounds. Generally, the potencies of inhibiting 11β-HSD1 activity for cyclic pentadienone analogues were significantly reduced (Tables 1), indicating that the different structures in the central spacer may play a role in the effects of 11β-HSD1. For example, compound **9** [(1E,4E)-1,5-bis(3-methylthiophen-2-yl) cyclopentanone] did not inhibit human and rat 11β-HSD1 at 100 μM, and compound **16** [(1E,4E)-1,5-bis(thiophen-2-yl) cyclohexanone] inhibited human 11β-HSD1 activity with reduced potency (IC_50_ = 3.57 μM) compared to the open chain pentadienone compound **6**, IC_50_ = 93 nM). There was also species-dependent inhibition, human 11β-HSD1 was more sensitive to the inhibition by compound **8** and **11** than rat one (Table 1).

**Table 1:** The potency data of curcumin analogues of inhibiting 11β-hydroxysteroid dehydrogenase 1 and 2 activities.

	Rat 11β-HSD1		Human 11β-HSD1		IC_50_ (nM) Rat	IC_50_ (nM) Human
	IC_50_ (nM) in Leydig cells	Potency to Curcumin (fold)	IC_50_ (nM) in CHOP cells	Potency to Curcumin (fold)	11β-HSD2	11β-HSD2
**1 (Curcumin)**	5,785	1.00	2,295	1.00	11,920	14,559
**2**	4,207	1.38	995	2.31	>100000	>100000
**4**	114	50.75	183	12.54	>100000	19,580
**6**	184	31.44	93	24.68	>100000	>100000
**7**	3,128	1.85	1,330	1.73	>100000	>100000
**8**	2,669	2.17	111	20.68	>100000	>100000
**9**	>100,000	NA	>100000	NA	>100000	>100000
**10**	>100,000	NA	>100000	NA	>100000	>100000
**11**	1,050	5.51	225	10.20	>100000	>100000
**12**	10,752	0.54	3,046	0.75	>100000	>100000
**13**	11,938	0.48	4,587	0.50	>100000	>100000
**15**	11,756	0.49	2,099	1.09	>100000	>100000
**16**	2,551	2.27	3,566	0.64	>100000	>100000

CHOP-11β-HSD1 = CHOP cells transfected with human *HSD11B1*; All potency data are reported as the mean of at least two determinations; NA = not available.

Curcumin was slightly selective against both human and rat 11β-HSD2 with IC_50_ values of 14.56 and 11.92 µM, respectively (Table 1). However, compound **6,** **8** and **11** were highly selective against human and rat 11β-HSD2 activity, since they did not inhibit human and rat 11β-HSD2 at all at 100 µM (Table 1). Compound **4** also inhibited human 11β-HSD2 activity with IC_50_ value of 19.58 µM.

### Mode of inhibition of curcumin derivatives

**Figure 4:**
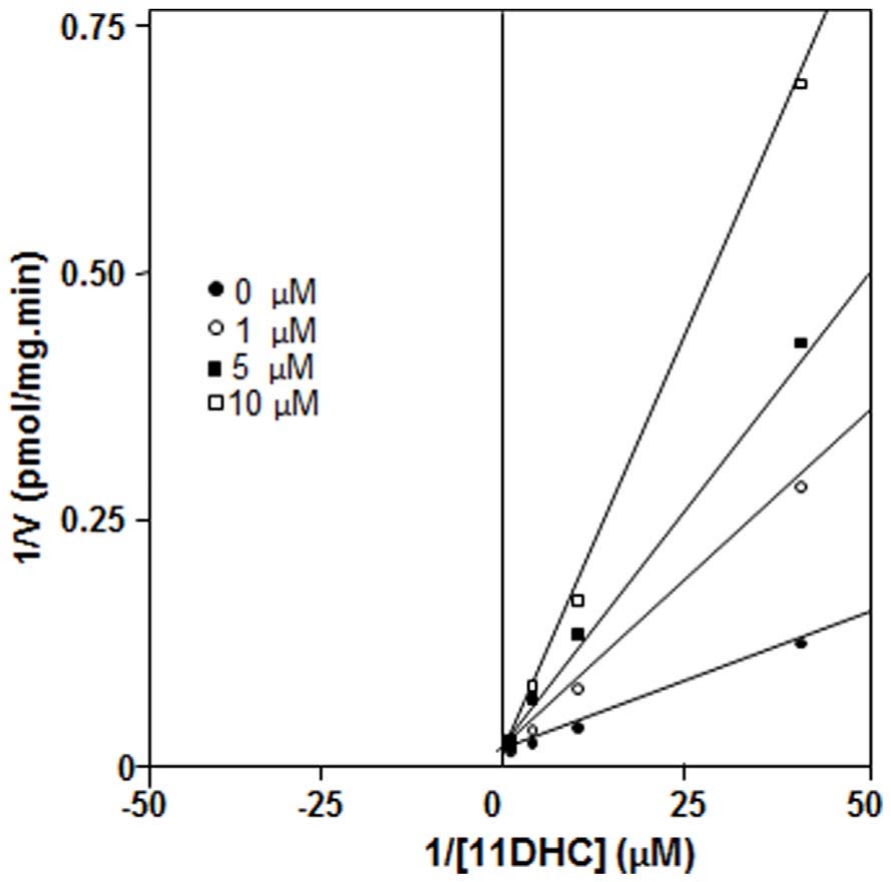
Lineweaver–Burk plot of rat liver microsomal 11β-HSD1 in the presence of compound 6. 1/V versus 1/[11DHC], V, velocity (pmol/mg protein⋅min); [11DHC], concentrations of 11DHC.

Using the compound **6** as an inhibitor to test the mode of inhibition of rat 11β-HSD1 activity, it was found that compound **6** inhibited rat 11β-HSD1 activity with a competitive mode (Fig. 4). This compound inhibited human liver 11β-HSD1 activity by the same mode (data not shown). Curcumin showed the similar mode to compound 6 as an inhibitor of 11β-HSD1 in both human and rat enzymes (data not shown).

### Physiological parameters, serum glucose and lipid profiles after curcumin treatment

**Figure 5:**
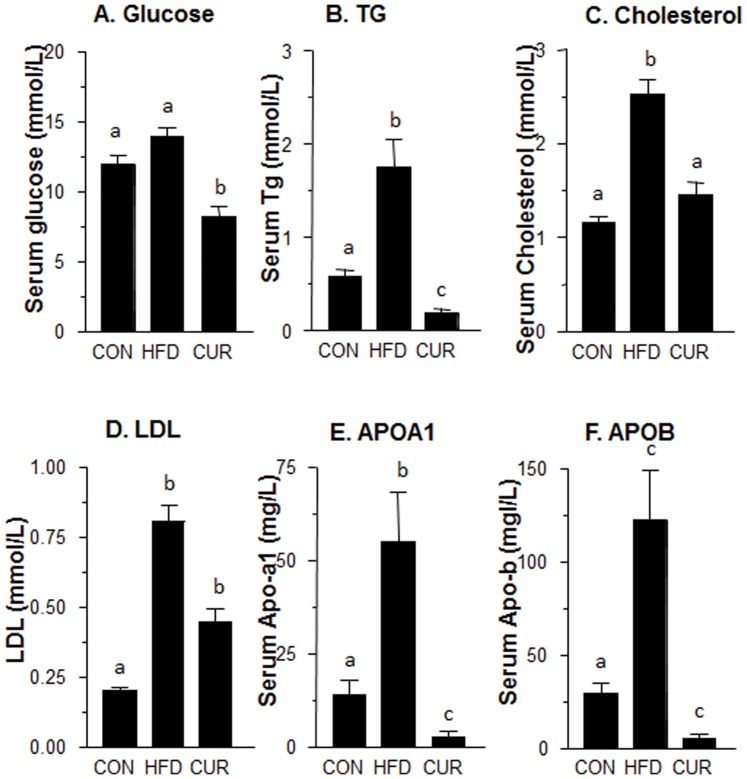
Serum glucose, total cholesterol, Tg, LDL, APOA1 and APOB in rats from normal diet control (CON), HFD diet (HFD) and HFD plus curcumin (CUR). Male rats were administered with 200 mg/kg curcumin for 2 months. Mean ± SE, n = 10. Identical letter designates no significant difference between two groups at P<0.05.

During the two-month treatment, there were no adverse effects found in HFD and curcumin treatment groups. When compared to control, HFD induced significant increase of body and liver weight (Table S1). Curcumin reduced HFD-induced increase of body weight gain and liver weight after two months of treatment. However, both HFD and curcumin did not affect testis and kidney weights (Table S1). As shown in Fig. 5, the levels of serum glucose did not significantly increase after feeding with HFD, while Tg, cholesterol, LDL, APO1A and APOB in HFD group were all significantly higher than those in control group (normal diet). Treatment with curcumin significantly lowered glucose, Tg, cholesterol, LDL, APO1A and APOB levels (Fig. 5), indicating that curcumin is a good compound to treat metabolic syndrome.

## Discussion

The present study demonstrates that curcumin and its derivatives are the selective inhibitors of 11β-HSD1. Curcumin had inhibitory effects on both human and rat 11β-HSD1 with IC_50_ values of about 2–6 µM when measured in intact cells. Some curcumin derivatives had higher potencies for inhibition of both human and rat 11β-HSD1 with IC_50_ values of about 100 nM. Apparently, administration of 200 mg/kg body weight curcumin effectively improved the lipid profiles and reduced serum glucose level in HFD-induced metabolic condition in the rat.

11β-HSD1 is abundantly expressed in liver and adipose tissues, where it activates GCs locally [11], [19], [20]. Elevation of GCs, such as in Cushing's syndrome, is closely associated with the pathogenesis of metabolic syndrome, including insulin resistance, central obesity, hyperglycaemia and dyslipidaemia [21]. Unlike Cushing's syndrome, many cases of metabolic syndrome actually do not have the elevated GCs in the circulation [22], intracellular increases of GCs in liver or adipose tissues after the activation by 11β-HSD1 have been proposed [22]. It is true that over-expression of 11β-HSD1 gene (*Hsd11b1*) in the fat tissue of mice caused central obesity, disturbed lipid profiles and insulin resistance [3]. Over-expression of *Hsd11b1* in mouse liver also caused insulin-resistance, hypertension and fatty liver without obesity [23]. In contrast, inactivation of GCs in the fat tissue by artificial over-expression of 11β-HSD2, which normally is not expressed in adipocytes, prevented HFD-induced obesity and the imbalance of energy expenditure [24]. In addition, *Hsd11b1* null mice were resistant to HFD-induced insulin resistance, obesity and dyslipidaemia [25]. Morbidly obese patients normally had higher expression of 11β-HSD1 in their fat tissue, which was associated with insulin resistance [26], [27]. Therefore, it is of a great interest to discover safe and potent selective 11β-HSD1 inhibitors to treat metabolic syndrome. In current study, we screened a nutraceutical library, and found that curcumin is a selective 11β-HSD1 inhibitor. Curcumin inhibited both human and rat 11β-HSD1 with IC_50_ values of 2.30 and 5.79 µM, respectively, when measured in intact cells. Selectively against 11β-HSD2, curcumin inhibited human and rat 11β-HSD2 with IC_50_ values of 14.56 and 11.92 µM, respectively.

Curcumin has been used a coloring and flavoring additive in many foods, and the consumption in a normal diet is at the rate of up to 100 mg/day by people [28]. Human studies indicate that curcumin is tolerated in large oral doses, as high as to 8,000 mg/day, without apparent toxicity [29]. The selective inhibition of 11βHSD1 by curcumin could be applied to treat metabolic syndrome. Indeed, in the present study, HFD-induced dyslipidemia in the rat was effectively prevented after oral administration of 200 mg/kg/day. Although HFD did not increase serum glucose level, the curcumin treatment also reduced serum glucose level (Fig. 5A). Apparently, many reports have been documented about curcumin in regard to its beneficial effects on metabolic disorders in various animal models. Several earlier studies conducted in rats demonstrated that curcumin lowered serum and liver cholesterol levels [30], [31]. In diabetic rats fed with either normal or high fat diet, curcumin also remarkably reduced serum cholesterol and Tg levels [32]. In genetically mutant db/db mice that display many features of metabolic syndrome, including hyperglycemia, insulin resistance and obesity, curcumin treatment significantly reduced their serum glucose levels, lowered the body weights, and corrected the insulin resistance [33]. Although many mechanisms of curcumin have been proposed for its effects on obesity and metabolic disorders, such as activation of peroxisome proliferator-activated receptor γ (PPARγ) [34], antioxidation [35], and suppression of p300 and nuclear factor-kappaB [36], the selective inhibition of 11β-HSD1 by curcumin could be another mechanism. Indeed, many selective 11β-HSD1 inhibitors have been tested to improve the metabolic conditions in animals and humans (see review [6], [37]).

Although curcumin is an effective and moderate inhibitor of 11β-HSD1, it is unstable and poor absorption when administered orally [29]. A clinical report showed that oral doses of up to 180 mg of curcumin failed to reach detectable serum level [38]. However, within 1 h of oral administration, higher doses of curcumin up to 8 g rendered its peak levels of about 0.5–2 µM [29], a concentration that was within its IC_50_ range of inhibiting 11β-HSD1 activity. In the present study, we also found that several curcumin derivatives were more potent in the inhibition of human 11β-HSD1 with IC_50_ values of around 100–200 nM. These chemicals are more selective, because they did not inhibit 11β-HSD2 at 100 µM at all. Unlike curcumin, these compounds are more metabolically stable because of mono-carbonyl group [9]. Especially, the compound 6 was the most potent in the inhibition of human 11β-HSD1 with IC_50_ value of 93 nM. It was very selective against 11β-HSD2, and did not inhibit 11β-HSD2 at all at 100 µM. Like curcumin, the compound is a competitive inhibitor of 11β-HSD1. Whether the compound is more effective than curcumin in the treatment of metabolic syndrome is worthy to be tested in the future. Many selective 11β-HSD1 inhibitors have been developed. Biovitrum first started the development of selective 11β-HSD1 inhibitors based on high throughput screening of compounds and found that some arylsulphonamidothiazoles were more selective 11β-HSD1 inhibitors. Several compounds, including BVT14226, which inhibits human enzyme with an IC_50_ value of 52 nM and mouse enzyme with an IC_50_ value of 246 nM, and BVT2733, which inhibits human enzyme with an IC_50_ value of 3.3 µM and mouse enzyme with an IC_50_ value of 96 nM.). They showed more than 200-fold selective over human 11β-HSD2. Indeed, BVT2733 was evaluated in vivo in the hyperglycemic KKAy mouse model, and the results demonstrated that the compound significantly lowered blood glucose level [39]. Merck also disclosed triazole compounds as selective inhibitors of 11β-HSD1. The compound MK544 inhibited human 11β-HSD1 inhibitor with an IC_50_ value of 7.8 nM (98 nM for mouse) with >450- or >100-fold selectivity over human or mouse 11β-HSD2 [40]. Compared to these compounds, our curcumin derivatives among the potent inhibitors within the nanomolar range.

There is a clear structure activity response (S.A.R) relationship. The newly characterized compound 4 and 6 possesses IC_50_ in mid-nanomolar range and an up to 24-fold increase efficacy compared to the parent compound curcumin. In addition, it is found that chemical derivatives of curcumin like compound 6 and 11 are the most selective ligands for 11β-HSD1, since at 100 µM they did not inhibit 11β-HSD2 at all. The enhanced activity and selectivity seems to be conferred by thiophenyl pentacyclic ring structure in compound 4, 6 and 11.

In conclusion, we described several novel curcumin derivatives as the selective 11β-HSD1 inhibitors. These compounds displayed greater activity on human and rat 11β-HSD1. Even with limited understanding of the mechanisms and tissue specificity, they could become novel therapeutic agents targeting on 11β-HSD1 for treatment of metabolic syndrome.

## Supporting Information

Table S1 **Adult rats were fed with normal chow or high fat diet (HFD) or HFD with curcumin (200 mg/kg/day) for 2 months.** The effects of curcumin on body weight, the weights of liver, testis and kidney were recorded. Mean ± SEM, n = 10. Identical letter represents no significant difference between two groups at P<0.05 for each parameter.
(DOC)
