# Supplementary material for: Curcumin as a Potent and Selective Inhibitor of 11β-Hydroxysteroid Dehydrogenase 1: Improving Lipid Profiles in High-Fat-Diet-Treated Rats
Source: PLoS One. 2013 Mar 22;8(3):e49976. doi: 10.1371/journal.pone.0049976 (PMC3606385; doi:10.1371/journal.pone.0049976)
Supplement: Table S1 — Adult rats were fed with normal chow or high fat diet (HFD) or HFD with curcumin (200 mg/kg/day) for 2 months. The effects of curcumin on body weight, the weights of liver, testis and kidney were recorded. Mean ± SEM, n = 10. Identical letter represents no significant difference between two groups at P<0.05 for each parameter. (DOC) [file pone.0049976.s001.doc]

Supplementary table 1. Physiological parameters after curcumin treatment

|  | CON | HFD | HFD + Curcumin |
| --- | --- | --- | --- |
| Body weight (g) | 286.3±7.779^a^ | 340.9±12.05^b^ | 324.8±6.614^b^ |
| Weight Gain(g) | 93.25±7.722^a^ | 340.9±12.05^b^ | 136.3±6.516^c^ |
| Testis(g) | 2.542±0.0621^a^ | 3.590±1.036^a^ | 2.664±0.1012^a^ |
| Liver(g) | 10.33±0.344^a^ | 14.20±0.395^b^ | 10.67±0.297^a^ |
| Kidney(g) | 1.167±0.0355^a^ | 1.260±0.0400^a^ | 1.309±0.0756^a^ |

*Note:* Identical letter represents no significant difference between two groups at P < 0.05.
